# HAFNI-enabled largescale platform for neuroimaging informatics (HELPNI)

**DOI:** 10.1007/s40708-015-0024-0

**Published:** 2015-11-27

**Authors:** Milad Makkie, Shijie Zhao, Xi Jiang, Jinglei Lv, Yu Zhao, Bao Ge, Xiang Li, Junwei Han, Tianming Liu

**Affiliations:** 1 Cortical Architecture Imaging and Discovery Lab, Department of Computer Science and Bioimaging Research Center, The University of Georgia, Athens, GA USA; 2 School of Automation, Northwestern Polytechnical University, Xi’an, China; 3 School of Physics & Information Technology, Shaanxi Normal University, Xi’an, China

**Keywords:** fMRI, Big data, Informatics system, HELPNI, HAFNI, XNAT

## Abstract

Tremendous efforts have thus been devoted on the establishment of functional MRI informatics systems that recruit a comprehensive collection of statistical/computational approaches for fMRI data analysis. However, the state-of-the-art fMRI informatics systems are especially designed for specific fMRI sessions or studies of which the data size is not really big, and thus has difficulty in handling fMRI ‘big data.’ Given the size of fMRI data are growing explosively recently due to the advancement of neuroimaging technologies, an effective and efficient fMRI informatics system which can process and analyze fMRI big data is much needed. To address this challenge, in this work, we introduce our newly developed informatics platform, namely, ‘HAFNI-enabled largescale platform for neuroimaging informatics (HELPNI).’ HELPNI implements our recently developed computational framework of sparse representation of whole-brain fMRI signals which is called holistic atlases of functional networks and interactions (HAFNI) for fMRI data analysis. HELPNI provides integrated solutions to archive and process large-scale fMRI data automatically and structurally, to extract and visualize meaningful results information from raw fMRI data, and to share open-access processed and raw data with other collaborators through web. We tested the proposed HELPNI platform using publicly available 1000 Functional Connectomes dataset including over 1200 subjects. We identified consistent and meaningful functional brain networks across individuals and populations based on resting state fMRI (rsfMRI) big data. Using efficient sampling module, the experimental results demonstrate that our HELPNI system has superior performance than other systems for large-scale fMRI data in terms of processing and storing the data and associated results much faster.

## Introduction

Understanding the organization of brain function has received significant interest since the establishment of neuroscience. During the past two decades, functional magnetic resonance imaging (fMRI), which is an in vivo neuroimaging technique, has revolutionized the functional mapping of the brain [1–8]. Specifically, task-based fMRI (tfMRI) has been widely used to record functional brain activities during a specific task performance and further to identify brain regions that are functionally involved in the task performance [2, 4, 5]. Meanwhile, resting state fMRI (rsfMRI) has also received intense interest more recently to acquire brain activities while participants are in a task-free state. The coherence in the functional brain organization which is free from the task performance constraint can be reflected based on the spontaneous signal changes during resting state [1, 3–8].

Given the importance of fMRI (including both tfMRI and rsfMRI) data for functional brain mapping, tremendous efforts have been devoted on the establishment of fMRI informatics systems which recruit a comprehensive collection of statistical/computational approaches for fMRI data analysis [9–14]. For example, MEDx is one of the earliest tools which was produced to incorporate advances in neuroimaging methods in 1993 [9]. Later on, FSL (FMRIB’s Software Library) toolbox was developed to bring more insights to the neuroscience analysis tools, and since June 2000, it has helped researchers globally apply FEAT, MELODIC, FABEER, BASIL, and VERBENA tools for fMRI data processing and analysis [10, 11]. Moreover, statistical methods and tools have become one of the main tools to study brain networks and connectivity. For example, statistical parametric mapping (SPM) is one of the most influential tools which has been designed for brain imaging data sequence analysis from different cohorts or time- series [12]. Analysis of functional neuroimages (AFNI) package is another tool to visualize and statistically analyze fMRI datasets [13]. Furthermore, some have dedicated their resources to create a concentrate database to index the context and content of the fMRI literature in a searchable fashion, considering the multidisciplinary nature of fMRI researches and thousands of investigators around the globe. Fox and Lancaster have discussed demands of such a system and proposed BrainMap to address required applications [14, 15]. Although significant successes have been achieved for these fMRI informatics systems [16, 17], a considerable limitation is that all of those state-of-the-art systems are especially designed for specific fMRI sessions or studies of which the data size is not really big. As a consequence, there is difficulty for those systems to preprocess, analyze, and visualize fMRI ‘big data’ simultaneously.

With the advancement of neuroimaging technologies, the size of fMRI data is growing explosively. Given the lack of a uniform resource center for fMRI data providers, researchers, and developers, neuroimaging informatics tools and resources clearinghouse (NITRC) were established in 2006 to facilitate finding and computing neuroimaging resources for functional and structural neuroimaging analyses to be a common place to share required tools and data [18]. Although it was not for the first time that a government-funded project became an international neuroscience resource provider to cover pioneers worldwide, for example, neuroscience information framework (NIF) in 2004 [19] as well as biomedical informatics research network (BIRN) in 2001 [20], NITRC was successful and popular to host and provide one of the biggest fMRI databases named 1000 functional connectomes (1000FC) resting state fMRI project. [https://www.nitrc.org/projects/fcon_1000/]. Moreover, there are other fMRI big datasets that are publicly available for researchers such as OpenfMRI [21] and human connectomes project (HCP) [22]. HCP is a recent NIH-funded project devised to map the brain’s communication network called connectome. This project provides a collection of neural data along with an interface to graphically navigate the data. The OpenfMRI is a National Science Foundation funded project established in 2010 to provide resources for researches to upload their owned fMRI data and make them publicly available.

In short, the availability of fMRI big data has globally attracted increasing attention for researchers in the neuroimaging field to test various methods and algorithms based on a ‘big data’ strategy. For instance, the velocity of studies as well as the variety and volumes of neuroimages is aggregating exponentially, which are among the biggest challenges nowadays [23]. As Van Horn studied and mentioned [24], the calculated neuroimaging data from listed articles in representative issues of neuroimage have been increased drastically and it is being expected to grow exponentially. The average size of raw data per study is expected to be 15 GB in 2015 and 20 GB in 2020. Therefore, effective and efficient fMRI informatics systems which can process and analyze fMRI big data are much needed.

**Fig. 1:**
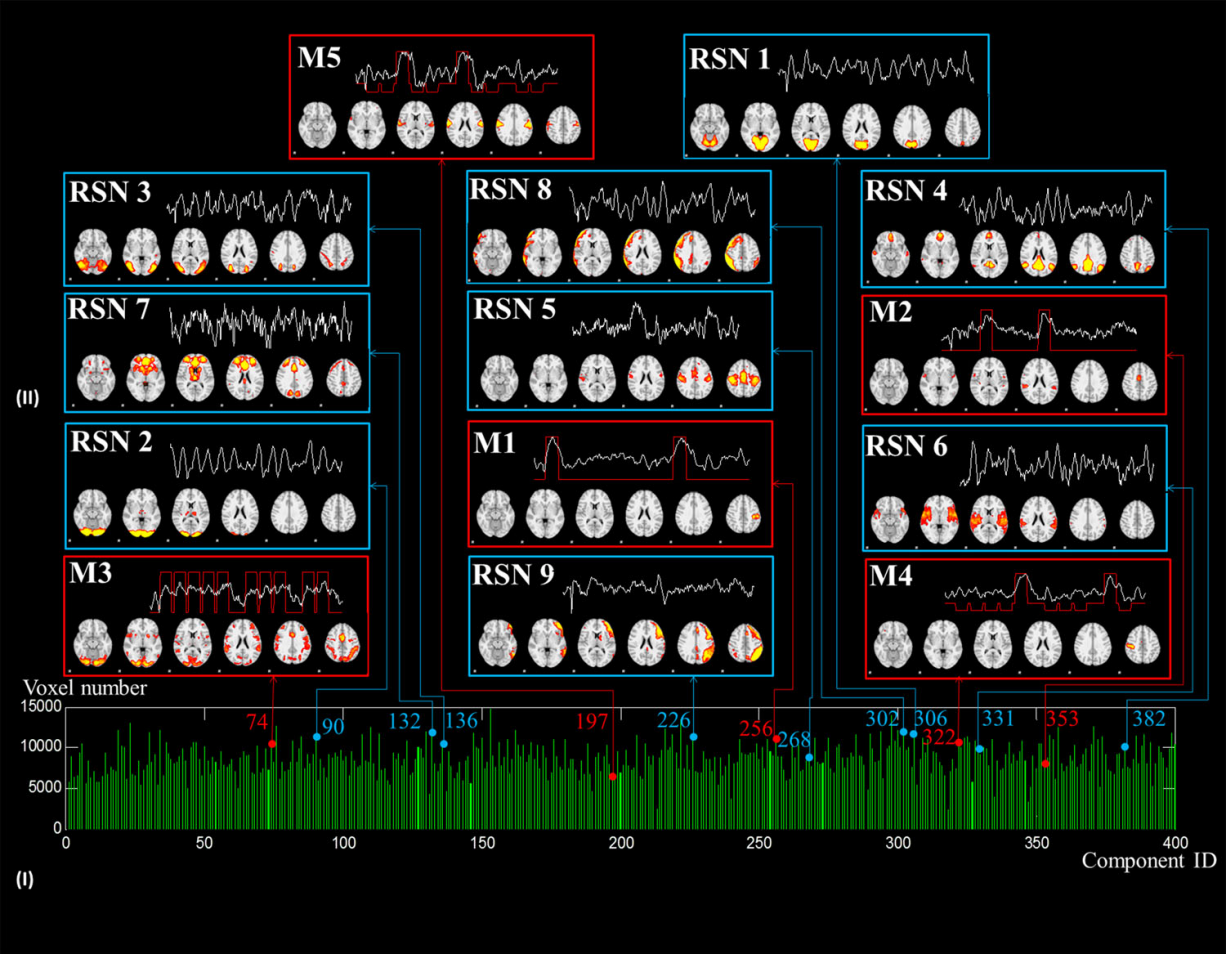
**I** The decomposed dictionary components from the fMRI data during one single task and **II** the 14 corresponding reference weight maps by applying the HAFNI method to the whole-brain fMRI signals. This figure visualizes 14 selected dictionary components which are either motor task-evoked networks (M1–M5) or resting state networks (RSN1–RSN9). The *green bars* in (**I**) show 400 dictionary network components (indexed along *x*-axis) and the spatial non-zero voxel numbers that each component’s reference weight map contains (represented by the *horizontal* height of each *bar*). The panels in (**II**) visualize the temporal time series (*white curve*) and spatial distribution map (eight representative volume images) of each network. The *red curves* represent the task contrast designs of the motor tfMRI data. (Color figure online)

To deal with the abovementioned limitation of previous fMRI informatics systems and to address the need of effective fMRI informatics system which can process and analyze fMRI big data for researches, in this paper, we have developed a HAFNI-enabled largescale platform for neuroimaging informatics (HELPNI) (http://bd.hafni.cs.uga.edu/helpni). This system is established using the extensible neuroimaging archive toolkit (XNAT) web application and storage solutions [25], a widely used open source system for storing, managing, and analyzing medical images and related meta-data [26]. RESTful application programming interface makes it especially useful for data sharing since the entire database’s contents are reachable programmatically through the web application [26]. Specifically, the proposed HELPNI system in this work implements our latest computational framework of sparse representation of whole-brain fMRI signals which is called ‘holistic atlases of functional networks and interactions’ (HAFNI) [27]. The main idea of HAFNI is to aggregate all of hundreds of thousands of tfMRI or rsfMRI signals within a whole brain of one subject into a big data matrix, which is subsequently factorized into an over-complete dictionary basis matrix (represented by the panel (I) of Fig. 1) and a reference weight matrix (represented by the panel (II) of Fig. 1) via an effective online dictionary learning algorithm [28, 29]. The time series of each over-completed basis dictionary represents the functional BOLD (blood-oxygen-level dependent) activities of a brain network (the white curves in the panel (II) of Fig. 1) and its corresponding reference weight vector stands for the spatial map of this brain network (the volume images in the panel (II) of Fig. 1). The HAFNI framework has been found to be effective and efficient in inferring a comprehensive collection of concurrent functional networks in the whole brain [27]. HELPNI covers the fMRI big data both from big data matrix and high volume of subjects. This happens first through employing HAFNI framework to handle the big data matrix for each subject and second by utilizing a database to store large-scale datasets, and then using a scheduling engine to distribute analyzing tasks to multiple machines and process multiple subjects simultaneously. HELPNI, as an advanced informatics system, provided us with resources to identify large-scale (over all 1200+) functional connectomes subjects automatically via automated computational pipeline based on our HAFNI framework function, to store the results in an organized data structure, and to generate detailed reports for data analysis (containing registration, online dictionary learning, and identified functional brain networks results) accessible through our web interface publicly. The HELPNI system significantly expands the previous neuroimaging archive toolkit by adding HAFNI capabilities, that is, HAFNI-enabled, while significantly enhancing HAFNI by integrating the advanced informatics system.

The rest of this paper is organized as follows. We will describe the methods of development in addition to obtained results of HAFNI implementation in Sect. 2. We will also discuss the significance of this system in comparison to the previous methods of fMRI analysis studies. Results are provided in Sect. 3, and discussion and conclusion are in Sect. 4.

## Method

In this section, we first provide a technical overview of HELPNI system and then we discuss HAFNI implementation details and its workflow in our system. Subsequently, we will discuss the 1000FC database we used as the test bed in this paper.

### Overview of HELPNI system

**Fig. 2:**
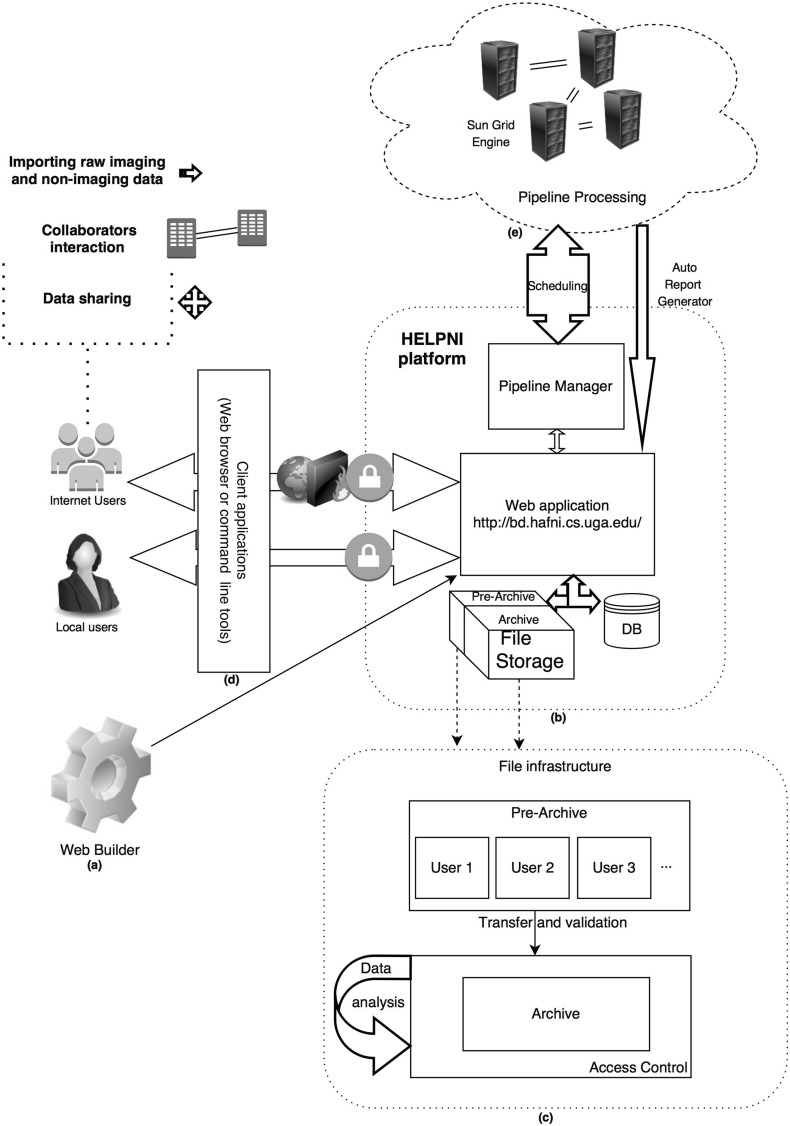
HELPNI structure and connected components. **a** Web builder through which the web application will be built. **b** HELPNI platform big picture. **c** File infrastructure workflow consists of pre-archive and archive in which data will be temporary stored and then after user inspection and running required processes, data will be moved to their permanent destination where pipelines processes will be run on. **d** Client application and users transactions. Local and global users connect to the web interface after logging into the system and passing firewall, using their preferred client application. Then, they will be able to process, share, download, and upload data interactively. **e** Pipeline processing unit(s) that dynamically receive parameters and executives from pipeline manager and after running predefined steps, generate a user friendly report along with required notifications and then will store the results into file storage

The main purpose of HELPNI is to store and manage large diverse imaging datasets to facilitate neuroimaging researches with complicated processes and large amount of data. The interesting feature of this platform is the extendibility, through which developers can customize their desired analytical and visualization tools. The platform uses XML schema to generate custom components, modules, workflows for different tiers. As Fig. 2 elaborates, the standardized workflow helps users to (a) capture imaging/non-imaging data and meta-data (either from neuroimaging machines or other databases manually); (b) inspect data by means of pre-archiving feature; (c) analyze data remotely or locally on-demand; (d) collaborate easier using the predefined filter (in this way, collaborators can be noticed when a related dataset were added to system); and (e) control access and share data where datasets and linked results can be shared publicly through the web interface to facilitate collaboration.

In the HELPNI system, we implemented our recently developed HAFNI framework for fMRI data analysis using the extendible pipelines. Pipeline is a workflow described in a XML document. Parameters could be specified within the XML document or be sourced as another XML document. So far we have implemented a few pipelines each of which contains different sets of scripts for our HAFNI framework. These pipelines can both extract input parameters from subjects automatically or ask users to provide them manually. Pipeline engine works based on the Java framework which parses parameters out of XML document and it links sequence of activities into a defined process flow and can manage data flow at each step. It can be configured to send notification at desired step(s) for quality control or to modify parameters, and then restart pipeline from where it stopped. We have used pipeline to automate the whole processes of fMRI data registration and online dictionary learning (ODL) and to reduce the processing time. It also helped to run the data over a very large set of data in much less amount of time as we implemented it over the 1000FC data. Pipelines can leverage from distributed computing, and in this way, a huge amount of processes can result in much less computation time.

**Fig. 3:**
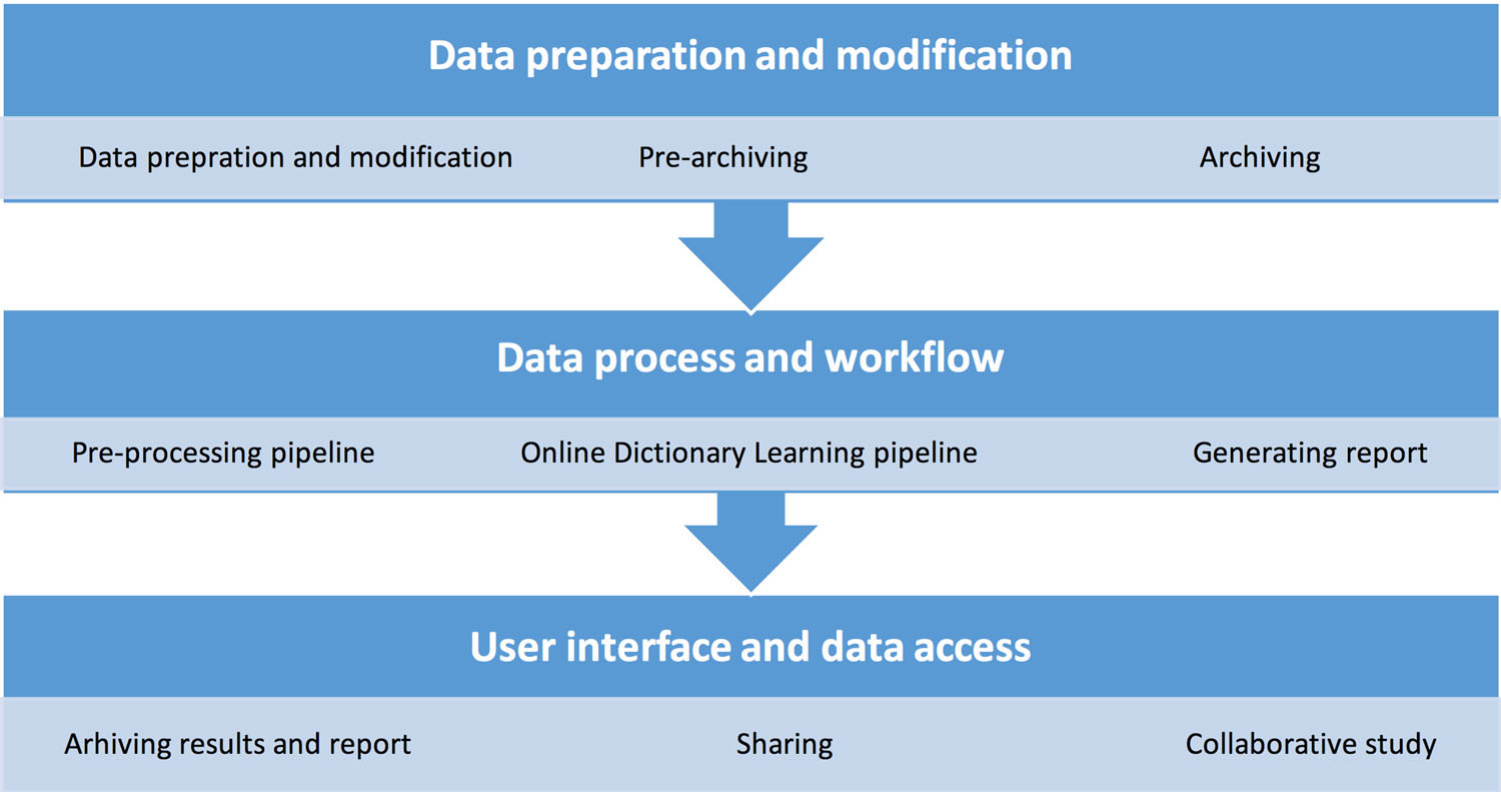
An overview of HAFNI implementation through HELPNI and its workflow

In this work, we used the 1000FC project datasets as test bed for HELPNI system developing and testing. The 1000FC project contains 1200+ resting state functional MRI (rsfMRI) images collected from 33 locations. We defined a workflow to obtain the result as we discuss here. Figure 3 shows the implemented pipelines and workflow of our process from the beginning of obtaining fMRI data from NITRC to data process steps and finally result reporting. The main three steps of this workflow are (a) data preparation and modification; (b) data process and workflow; and (c) user interface and data access as detailed in Sects. 2.2 and 2.3, respectively.

### Data preparation and modification

At the very first step, users need to prepare data to import to system. We first obtained data from 1000FC database and modified the data structure as our own predefined structure. After modifying hierarchy and trimming data, images with correspondent meta-data should be uploaded to pre-archive for primary tests and analysis. The required format of data should be created in file system including ID and sequence type as well as any special data type that needs to be defined in system. To do so, we prepared required meta-data including TR value, field strength, gender, and handedness of each subject and experiment. Then, data were transferred to pre-archive as a temporary cache destination for further tests and review of quality (Fig. 2c). Pre-archiving step keeps data integrated and protects them from data loss or corruption. We also tested our workflow to fix any possible flaw in implemented algorithms. When data became ready and analytical methods turn mature to be modeled in XML schema, we imported data into the archive as final destination for viewing purposes and/or running standard processes on prepared data. We used curl to upload fMRI data through REST API [30] from command line.

### Data process and workflow

The next step in HELPNI platform is data processing. The raw fMRI data need to be preprocessed before data analysis. We implemented the rsfMRI and tfMRI preprocessing pipeline in HELPNI to address this demand. Our preprocessing step includes skull removal, motion correction, slice time correction, and special smoothing as well as global drift removal [8]. We used *Build* and *ArcBuild* [26] predefined XNAT tools for image session scan selection and running processing steps, respectively.

Applying the major processing pipeline is the next step. We integrated our HAFNI computational framework in HELPNI. The basic idea of HAFNI framework [27] is to aggregate all of the thousands of fMRI signals within the whole brain from one subject into a big data matrix and then decomposes it into an over-completed dictionary matrix and a reference coefficient matrix. Specifically, each column of the dictionary matrix represents a typical brain activity pattern and the corresponding row in coefficient matrix naturally reveals the spatial distribution of the activity pattern. Typically, each subject brain’s signals form an *m* × *n* matrix *S*, with *m* represents the fMRI time points (observations) and *n* represents the number of voxels. In order to sparse represent the signal matrix *S* using *D*, we aimed to learn a meaningful and over-completed dictionary matrix $D{ \in {\mathbb{R}}}^{m \times k}$ (*k* > *m, k* << * n*), with *k* being the dictionary atoms (i.e., components). The loss function is defined in Eq. (1) with a $l_{1}$ regularization that yields to a sparse resolution of $\alpha_{i}.$

1 $$ l\left( {s_{i} ,D} \right) \triangleq \mathop {\hbox{min} }\limits_{{\alpha_{i} { \in {\mathbb{R}}}^{m} }} \frac{1}{2}||s_{i} - D\alpha_{i} ||_{2}^{2} + \lambda ||\alpha_{i} ||_{1} $$

Here $\alpha_{i}$ is the coefficient matrix and *λ* is a sparsity regularization parameter. In order to prevent *D* from arbitrarily large values, the columns $d_{1} ,d_{2} , \ldots d_{m}$ are constrained by Eq. (2).

2 $$ C \triangleq \left\{ {D{ \in }{\mathbb{R}}^{t \times m}    \quad {\text{s.t.}} \quad \forall j = 1, \ldots m,  d_{j}^{T} d_{j} \le 1} \right\} $$

3 $$ \mathop {\hbox{min} }\limits_{{D{ \in }C,\alpha { \in {\mathbb{R}}}^{m \times n}  }} \frac{1}{2}||S - D\alpha ||_{F}^{2} + \lambda ||\alpha ||_{1 } $$

Briefly, the problem can be transferred into a matrix factorization problem in Eq. (3) and we adopted the state-of-the-art online dictionary learning algorithm [29] for the sparse representation of the whole-brain fMRI signals.

**Fig. 4:**
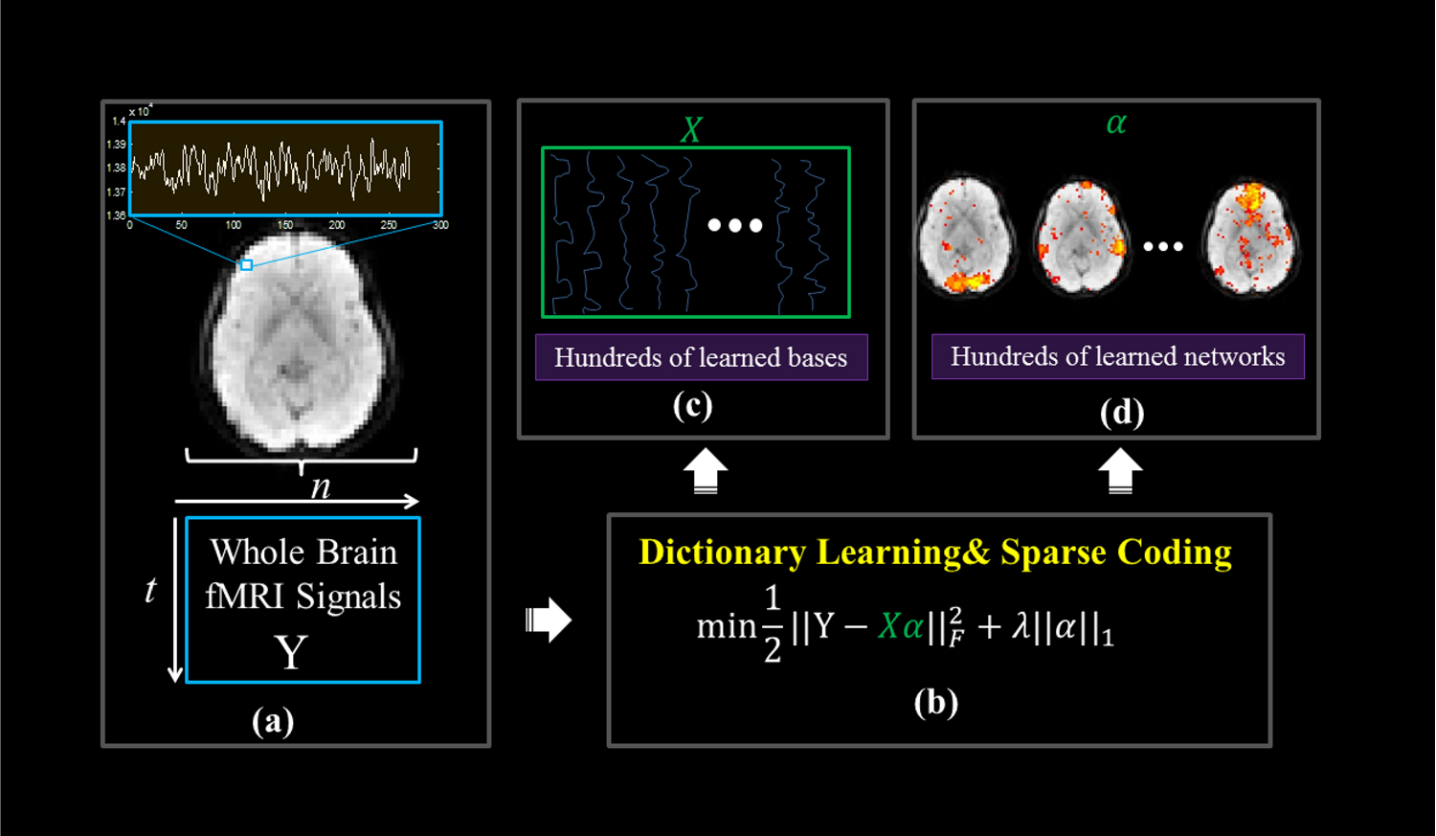
The computational pipeline of sparse representation of whole-brain fMRI signals using an online dictionary learning approach. **a** The whole-brain fMRI signals are aggregated into a big data matrix, in which each row represents the whole-brain fMRI BOLD data in one time point and each column contains the time series of one single voxel. **b** The target optimization function of dictionary learning and sparse coding. **c** Illustration of the learned atomic dictionary, each dictionary represents one functional network component. **d** The coefficient matrix, each row in the matrix measures the weight coefficient of the corresponding dictionary over the whole brain. That is, each row defines the contribution of one dictionary to the composition of all voxel-wise fMRI signals

Once we obtained the learned dictionary matrix D and coefficient matrix $\alpha$, we mapped each row in the *α* matrix back to the brain volume and examine their spatial distribution patterns, through which functional network components are characterized on brain volumes [27]. At the conceptual level, the sparse representation framework in Fig. 4 can achieve both compact high-fidelity representation of the whole-brain fMRI signals (Fig. 4c) and effective extraction of meaningful patterns (Fig. 4d) [28, 29, 31–34]. For more details, please refer to our recent literature report [27].

The system is designed to feed the preprocessing as the input of online dictionary learning pipeline automatically or manually after filtering the preprocessed data. For visualization purposes and to make the generated results easy to explore, both preprocessing and ODL pipelines will generate a PDF report at the end after which it will be automatically uploaded to the web interface. These reports contain generated results from the executed pipelines identified by experiment ID appended to pipeline name. For example, ODL report contains 400 png files sorted sequentially.

Pipelines can also be set to send notification within different steps of workflow. For example, user can be notified when a specific step is done to evaluate the result and then if it meets the quality, let the pipeline continue. Otherwise, user can modify the input variables and restart the pipeline. Also at the end of workflow, assigned users will be notified of a successful run.

### User interface and data access

Large-scale fMRI data usually need group-wise analysis and collaborators need to work together. In HELPNI, users can connect to system remotely and choose their desired subset of archive through bundle feature in the system. Users are also able to email other collaborators a link containing selected subset of archive.

The standard user interface features useful tools including a search box which provides searching through all archived subjects and sessions and menus in which users upon their permissions can access. Users need to login to system to be able to modify or upload new data but viewing and downloading 1000FC data as well as preprocessing and ODL results are publicly available (http://bd.hafni.cs.uga.edu/helpni). User can browse experiments and data via three methods. One is by selecting project and subject subsequently, and the other is through searching for a subject name from search box, and the last is through selecting a listing, where user can input certain information of project/subject or image modality and then query a list containing correspondent filtered data.

## Results

We tested the proposed HELPNI platform by applying the implemented computational framework of HAFNI on one of the largest open source resting state fMRI (rsfMRI) databases: 1000 Functional Connectomes project (known as 1000FC) [6]. This database has gathered more than 1200 rsfMRI datasets independently collected from all over the world containing over 130 Giga Bytes of data. Table 1 (see http://fcon-1000.projects.nitrc.org) summarizes rsfMRI datasets. Age, sex, and imaging center information are provided for each of datasets and all subjects have been uploaded to the HELPNI. As detailed in Sect. 2, HELPNI automatically preprocessed the raw rsfMRI data, extracted the rsfMRI signals from each subject, applied the HAFNI computational framework, and returned and stored meaningful experimental results. In this experiment, we used 8-core Intel^®^ Xeon^®^ E5-2650 v2 2.60 GHz, 20 M Cache CPU and 32 GB RDIMM, 1600MT RAM. With the help of HELPNI, we identified consistent and meaningful functional brain networks across individuals and populations based on rsfMRI big data which are detailed in Sect. 3.1. Moreover, using HELPNI possess modularity and plug-and-play capability, we developed an efficient sampling module and integrated it with HAFNI framework to speed up the HAFNI overall computational time and to automatically calculate and obtain meaningful functional brain networks in a much faster fashion. The detailed results are demonstrated in Sect. 3.2.

**Table 1:** The 1000 Functional Connectomes Project datasets summary

Baltimore (*n* = 23 [8 M/15F]; ages: 20–40; TR = 2.5; # slices = 47; # timepoints = 123)	Bangor (*n* = 20 [20 M/0F]; ages: 19–38; TR = 2; # slices = 34; # timepoints = 265)	Beijing_Zang (*n* = 198 [76 M/122F]; ages: 18–26; TR = 2; # slices = 33; # timepoints = 225)	Berlin_Margulies (*n* = 26 [13 M/13F]; ages: 23–44; TR = 2.3; # slices = 34; # timepoints = 195)
Cambridge_Buckner (*n* = 198 [75 M/123F]; ages: 18–30; TR = 3; # slices = 47; # timepoints = 119)	Cleveland CCF (*n* = 31 [11 M/20F]; ages: 24–60; TR = 2.8; # slices = 31; # timepoints = 127)	Dallas (*n* = 24 [12 M/12F]; ages: 20–71; TR = 2; # slices = 31; # timepoints = 115)	Durham_Madden (*n* = 42 [n/a]; ages: n/a; TR = n/a; # slices = n/a; X timepoints = n/a)
ICBM (*n* = 86 [41 M/45F]; ages: 19–85; TR = 2; # slices = 23; # timepoints = 128)	Leiden_2180 (*n* = 12 [12 M/0F]; ages: 20–27; TR = 2.18; # slices = 38; # timepoints = 215)	Leiden_2200 (*n* = 19 [11 M/8F]; ages: 18–28; TR = 2.2; # slices = 38; # timepoints = 215)	Leipzig (*n* = 37 [16 M/21F]; ages: 20–42; TR = 2.3; # slices = 34; # timepoints = 195)
Milwaukee_a (*n* = 18 [n/a]; ages: n/a; TR = 2; # slices = 20; # timepoints = 175)	Milwaukee_b (*n* = 46 [15 M/31F]; ages: 44–65; TR = 2; # slices = 64; # timepoints = 175)	Munchen (*n* = 16 [10 M/6F]; ages: 63–73; TR = 3; # slices = 33; # timepoints = 72)	Newark (*n* = 19 [9 M/10F]; ages: 21–39; TR = 2; # slices = 32; # timepoints = 135)
NewHaven_a (*n* = 19 [10 M/9F]; ages: 18–48; TR = 1; # slices = 16; # timepoints = 249)	NewHaven_b (*n* = 16 [8 M/8F]; ages: 18–42; TR = 1.5; # slices = 22; # timepoints = 181)	NewYork_a_ADHD (*n* = 25 [19 M/4F]; ages: 20–50; TR = 2; # slices = 39; # timepoints = 192)	NewYork_a (*n* = 84 [43 M/41F]; ages: 7–49; TR = 2; # slices = 39; # timepoints = 192)
NewYork_b (*n* = 20 [8 M/12F]; ages: 18–46; TR = 2; # slices = 33; # timepoints = 175)	NewYork_Test-Retest_Reliability (*n* = 25 [10 M/15F]; ages: 22–49; TR = 2; # slices = 39; # timepoints = 197)	Ontario (*n* = 11 [n/a]; ages: n/a; TR = 3; # slices = 29; # timepoints = 105)	Orangeburg (*n* = 20 [15 M/5F]; ages: 20–55; TR = 2; # slices = 22; # timepoints = 165)
Oulu (*n* = 103 [37 M/66F]; ages: 20–23; TR = 1.8; # slices = 28; # timepoints = 245)	Oxford (*n* = 22 [12 M/10F]; ages: 20–35; TR = 2; # slices = 34; # timepoints = 175)	PaloAlto (*n* = 17 [2 M/15F]; ages: 22–46; TR = 2; # slices = 29; # timepoints = 235)	Pittsburgh (*n* = 17 [10 M/7F]; ages: 25–54; TR = 1.5; # slices = 29; # timepoints = 275)
Queensland (*n* = 19 [11 M/8F]; ages: 20–34; TR = 2.1; # slices = 36; # timepoints = 190)	SaintLouis (*n* = 31 [14 M/17F]; ages: 21–29; TR = 2.5; # slices = 32; # timepoints = 127)	Taipei_a (*n* = 14 [n/a]; ages: n/a; TR = 2; # slices = 32; # timepoints = 295)	Taipei_b (*n* = 8 [n/a]; ages: n/a; TR = 2; # slices = 33; # timepoints = 175)
Atlanta (ages: 22–57; TR = 2; # slices = 20; # timepoints = 205)	AnnArbor_a (*n* = 25 [22 M/3F]; ages: 13–40; TR = 2; # slices = 40; # timepoints = 295)	AnnArbor_b (*n* = 36 [17 M/19F]; ages: 19–80; TR = 0.75; # slices = 16; # timepoints = 395)	

### Group-wise consistent functional brain networks identification using HELPNI

**Fig. 5:**
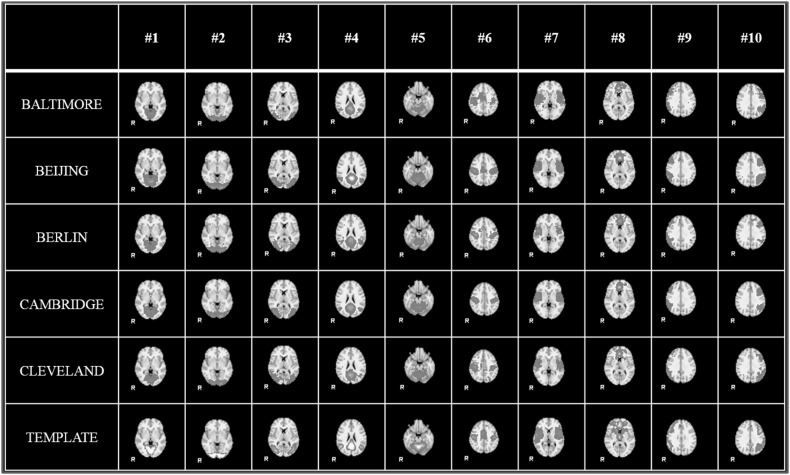
The identified group-wise consistent 10 RSN networks from 5 randomly selected datasets (Baltimore, Beijing, Berlin, Cambridge, and Cleveland) in 1000 Functional Connectomes Project by HELPNI. Each row represents the networks from one dataset; the last row shows the RSN templates for comparison. Only the most informative slice, which has been overlaid on the MNI152 template, is shown here

**Fig. 6:**
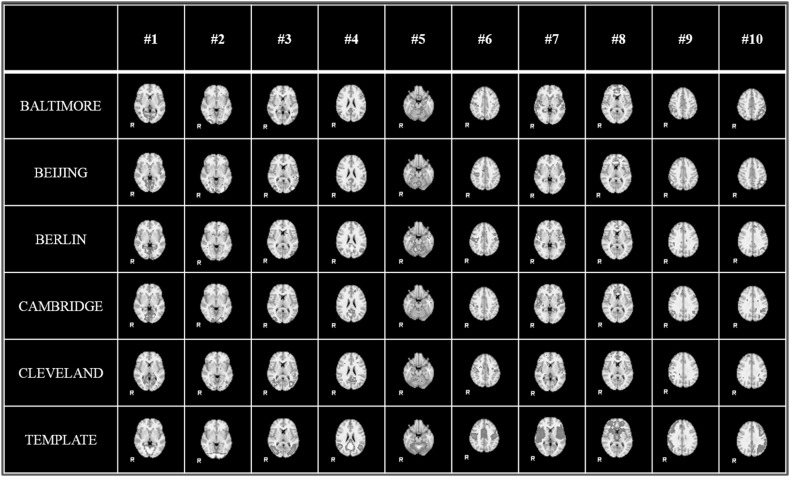
The identified 10 RSN networks of individual subject from 5 datasets (Baltimore, Beijing, Berlin, Cambridge, and Cleveland) in 1000 Functional Connectomes Project by HELPNI. For each dataset, the 10 RSN networks from one randomly selected subject are shown here

With the help of HELPNI system and the implemented HAFNI computational framework, we successfully identified 10 meaningful and consistent resting state networks (RSNs) which are in agreement with previous studies across all individuals and datasets in 1000FC database. Figure 5 shows the identified 10 group-wise consistent networks in five randomly selected datasets (that are Baltimore, Beijing, Berlin, Cambridge, and Cleveland dataset) in 1000FC. Networks #1, #2, and #3 are all located in visual areas and closely related to visual behavior. Network #4 includes ventromedial frontal cortex, bilateral inferior-lateral-parietal, and medial parietal areas and are often referred as default mode network (DMN). Network #5 covers the cerebellum and corresponds to action-execution function. Networks #6, #7, and #8 are related to sensorimotor, auditory, and executive control function, respectively. Networks #9 and #10 cover several front parietal areas and are closely related to cognition/language paradigms [35]. Figure 6 illustrates the identified 10 consistent networks in five randomly selected individual subjects from the same five datasets. We can see that the identified 10 functional networks are quite consistent across different datasets and subjects and consistent with the templates in previous studies [35]. Quantitatively, we calculate the spatial overlap between the identified networks and templates which are detailed in Table 2 and Table 3. The spatial overlap is calculated as the percentage of the overlapping area between our identified networks and templates [27]. Based on these results, we can see that our developed HELPNI system is effective and efficient in reconstructing meaningful functional brain networks from rsfMRI data.

**Table 2:** Spatial overlap between identified group-wise RSNs and templates in different datasets

	#1	#2	#3	#4	#5	#6	#7	#8	#9	#10
Baltimore	0.88	0.94	0.82	0.74	0.75	0.78	0.65	0.61	0.67	0.71
Beijing	0.95	0.98	0.95	0.82	0.86	0.94	0.85	0.58	0.66	0.82
Berlin	0.81	0.95	0.86	0.80	0.72	0.77	0.71	0.60	0.73	0.82
Cambridge	0.86	0.98	0.92	0.76	0.93	0.79	0.80	0.56	0.69	0.78
Cleveland	0.82	0.89	0.80	0.77	0.72	0.75	0.72	0.58	0.53	0.75

**Table 3:** Spatial overlap between identified individual RSNs and templates in different datasets

	#1	#2	#3	#4	#5	#6	#7	#8	#9	#10
Baltimore	0.34 ± 0.09	0.28 ± 0.09	0.29 ± 0.09	0.33 ± 0.05	0.23 ± 0.05	0.30 ± 0.07	0.21 ± 0.06	0.24 ± 0.05	0.21 ± 0.05	0.23 ± 0.06
Beijing	0.36 ± 0.09	0.29 ± 0.12	0.32 ± 0.12	0.37 ± 0.08	0.28 ± 0.09	0.41 ± 0.10	0.25 ± 0.07	0.27 ± 0.08	0.24 ± 0.06	0.26 ± 0.06
Berlin	0.32 ± 0.06	0.29 ± 0.09	0.24 ± 0.10	0.33 ± 0.06	0.23 ± 0.07	0.36 ± 0.09	0.25 ± 0.06	0.26 ± 0.05	0.27 ± 0.08	0.26 ± 0.05
Cambridge	0.35 ± 0.08	0.32 ± 0.10	0.33 ± 0.12	0.35 ± 0.07	0.41 ± 0.10	0.40 ± 0.09	0.25 ± 0.06	0.29 ± 0.05	0.23 ± 0.05	0.24 ± 0.05
Cleveland	0.32 ± 0.09	0.27 ± 0.13	0.25 ± 0.11	0.35 ± 0.06	0.19 ± 0.08	0.36 ± 0.09	0.22 ± 0.06	0.27 ± 0.06	0.24 ± 0.06	0.22 ± 0.05

### Integrating sampling module in HELPNI

**Fig. 7:**
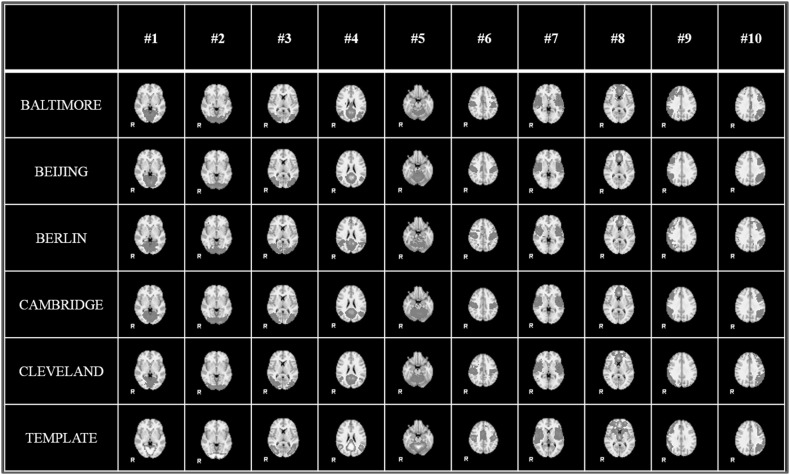
The identified group-wise consistent 10 RSN networks from 5 datasets (Baltimore, Beijing, Berlin, Cambridge, and Cleveland) in 1000 Functional Connectomes Project by HELPNI with sampling module. *Each row* shows the networks from one dataset and the last row shows the RSN templates for comparison

**Fig. 8:**
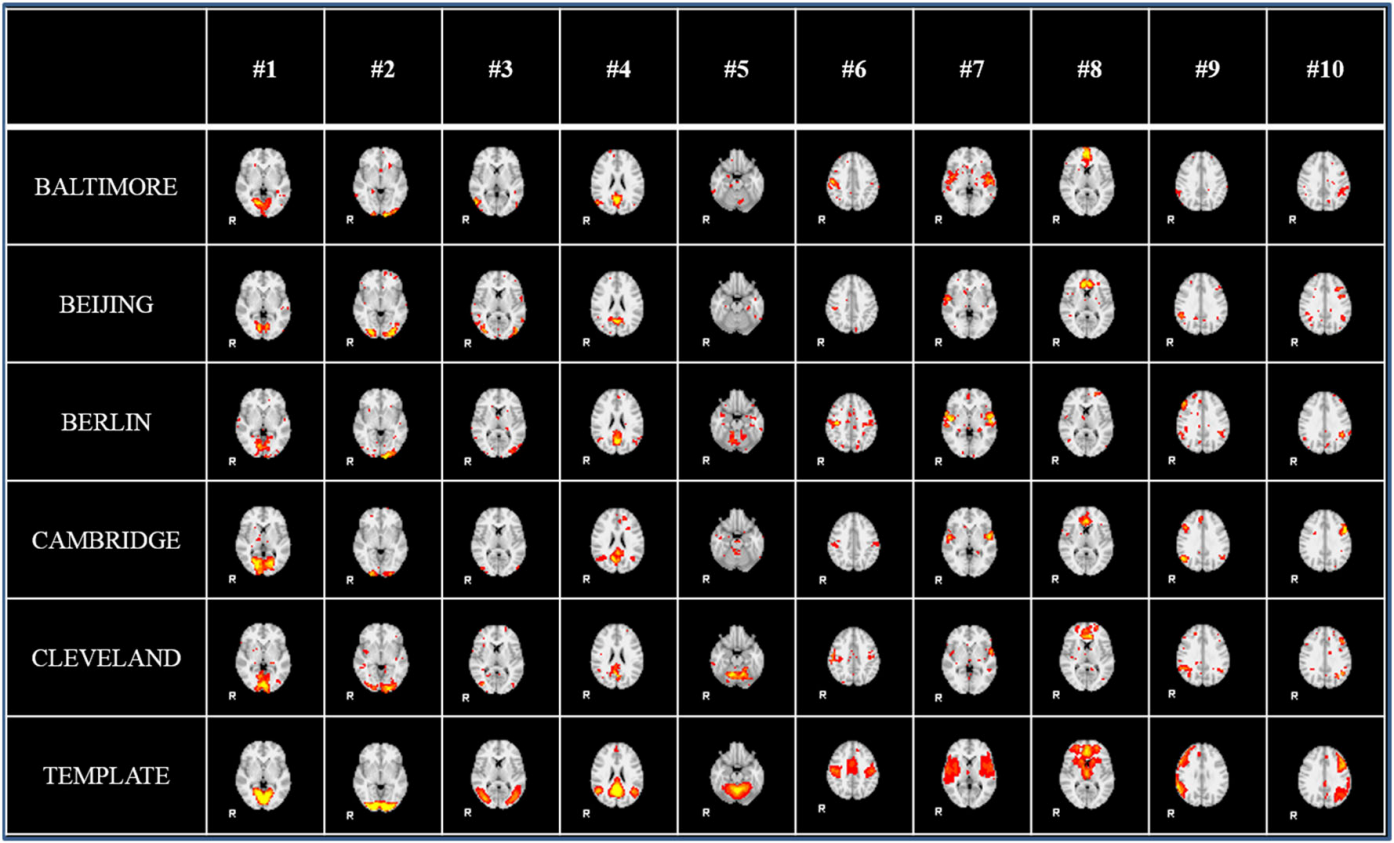
The identified 10 RSN networks of individual subject from 5 datasets (Baltimore, Beijing, Berlin, Cambridge, and Cleveland) in 1000 Functional Connectomes Project by HELPNI with sampling module. For each dataset, we randomly selected one subject’s result as example

One important characteristics of our HELPNI system is the plug-and-play capability. Since the implemented pipelines are modularly designed, we could easily develop and test new modules to enhance established computational framework. For example, in order to speed up the current HAFNI framework in the HELPNI system, we developed and integrated an efficient signal sampling module [36] to improve the calculating speed while obtaining comparable results. The average computation time of training a dictionary for one individual brain is about 30 s using sampling module, whereas the time cost without sampling is 340 s, which speeds up the HAFNI training procedure more than 10 times. At the same time, the returned results could identify the similar consistent and meaningful functional brain networks across datasets and individuals as discussed in Sect. 3.1. Figure 7 shows the same identified 10 group-wise consistent networks with sampling module in the same five datasets (that is Baltimore, Beijing, Berlin, Cambridge, and Cleveland dataset) in 1000FC. Figure 8 illustrates the identified 10 consistent networks in the same five individual subjects in Sect. 3.1. Similar to original HAFNI computational framework with no sampling module, the identified 10 functional networks are quite consistent with each other across different datasets and populations and consistent with the templates in previous studies [35]. Quantitatively, we calculated the spatial overlap between the identified networks and templates which are detailed in Tables 4 and 5. From these results, we can see that the integrated sampling module in HAFNI framework via HELPNI system significantly decreased the computing time while achieved comparable results for functional brain network identification at the same time. It also demonstrates the plug-and-play capability of HELPNI system to effectively detect meaningful functional brain networks from raw neuroimaging data.

**Table 4:** Spatial overlap between identified group-wise RSNs with sampling module and templates in different datasets

	#1	#2	#3	#4	#5	#6	#7	#8	#9	#10
Baltimore	0.89	0.89	0.82	0.79	0.76	0.92	0.64	0.59	0.68	0.72
Beijing	0.94	1.00	0.95	0.89	0.88	0.97	0.88	0.63	0.74	0.87
Berlin	0.87	0.95	0.90	0.83	0.73	0.87	0.76	0.68	0.88	0.82
Cambridge	0.84	0.98	0.94	0.84	0.95	0.86	0.82	0.57	0.68	0.83
Cleveland	0.80	0.95	0.88	0.82	0.75	0.75	0.77	0.61	0.57	0.74

**Table 5:** Spatial overlap between identified individual RSNs with sampling module and templates in different datasets

	#1	#2	#3	#4	#5	#6	#7	#8	#9	#10
Baltimore	0.38 ± 0.09	0.30 ± 0.10	0.29 ± 0.10	0.35 ± 0.06	0.26 ± 0.06	0.36 ± 0.08	0.21 ± 0.06	0.29 ± 0.07	0.24 ± 0.07	0.25 ± 0.06
Beijing	0.39 ± 0.11	0.32 ± 0.13	0.34 ± 0.13	0.39 ± 0.09	0.31 ± 0.10	0.43 ± 0.11	0.29 ± 0.08	0.31 ± 0.10	0.27 ± 0.07	0.29 ± 0.08
Berlin	0.36 ± 0.06	0.31 ± 0.10	0.28 ± 0.12	0.36 ± 0.08	0.24 ± 0.07	0.39 ± 0.08	0.26 ± 0.06	0.32 ± 0.05	0.28 ± 0.07	0.28 ± 0.06
Cambridge	0.37 ± 0.08	0.34 ± 0.11	0.33 ± 0.12	0.37 ± 0.07	0.44 ± 0.12	0.41 ± 0.09	0.27 ± 0.06	0.32 ± 0.05	0.26 ± 0.06	0.26 ± 0.06
Cleveland	0.34 ± 0.11	0.29 ± 0.13	0.25 ± 0.11	0.36 ± 0.05	0.20 ± 0.08	0.38 ± 0.08	0.24 ± 0.06	0.32 ± 0.08	0.26 ± 0.07	0.24 ± 0.06

## Discussion and conclusion

In this work, we have designed and developed a neuroimaging informatics platform, HELPNI, to archive large-scale fMRI datasets, to automate sequence of complex processes for fMRI data analysis and finally to use distributed and parallel computing resources to bust up big data analysis time. HELPNI has leverage from extensible neuroimaging archive toolkit to power up the web application and storage part of the system and is composed of three main parts of web application and storage, pipeline analysis framework, and the big data analytic tools. This novel platform integrated our recently developed HAFNI computational framework for fMRI data analysis in an accelerated way. As demonstrated in this work, we used the open access 1000 functional connectome datasets as a basic example to import 1200+ rsfMRI data into HELPNI system, to run the HAFNI framework on the rsfMRI data, and to identify consistent and meaningful functional brain networks across individuals and populations. Our experimental results demonstrated that efficient sampling module can be implemented together with HAFNI framework to speed up the dictionary learning and identification of meaningful functional brain networks.

The HELPNI platform is publicly accessible through http://bd.hafni.cs.uga.edu/helpni where users can view all of the archived fMRI data as well as the processed results. Authorized users can also upload new data and run pipelines over their desired fMRI images.

Considering the explained characteristics (Sect. 2) as well as the task scheduling feature of our HELPNI (Fig. 3e) in which tasks can be run in a distributed or parallel fashion, HELPNI with plug-and-play capability and modularity can significantly speed up the fMRI data processing. Users can easily feed their workflow to the HELPNI and it will schedule, distribute, and run all tasks using all available resources and will notify users with the final results. We are also implementing big data analytic tools to empower the processing part through Hadoop and Spark. Parallel optimization procedure has shown significant improvement in sparse dictionary learning computation time [37].

The large-scale datasets can be imported to the HELPNI system and various computational pipelines, and analyses can be then run over the big data without corrupting the original archived images. For example, in this paper, we ran the HAFNI pipeline over all subjects in 1000FC project, and the users could examine the results in a well-structured report in addition to original image data. We also ran the sampling pipeline on a subset of the dataset and stored them in the same fashion. In this way, users can evaluate and compare the results with sampling and no sampling simultaneously. The HELPNI system saved much computing time since there was no idle time in between of processes using the task scheduling feature. In the future, the distributed scheduling and big data analytics tools are planning to be used to save more time by means of distributed system available at the University of Georgia. This will provide fMRI community to use HELPNI system integrated with other analytical tools on large-scale fMRI datasets and to collaborate with other laboratories and research centers.

Adding a few new features including auto classifying the stored images based on the analysis results, fully implementing the parallel algorithm for HAFNI and improve the current user interface of HELPNI are scheduled as our future improvements to HELPNI. Future applications of HELPNI include testing other big datasets such as HCP and OpenfMRI, implementing new modules such as population clustering of learned dictionary HAFNI spatial maps, and eventually discovering disease-specific biomarkers.
